# Duration of labor and the risk of severe postpartum hemorrhage: A case-control study

**DOI:** 10.1371/journal.pone.0175306

**Published:** 2017-04-06

**Authors:** Lill Trine Nyfløt, Babill Stray-Pedersen, Lisa Forsén, Siri Vangen

**Affiliations:** 1 Division of Gynecology and Obstetrics, Oslo University Hospital, Oslo, Norway; 2 Institute of Clinical Medicine, Faculty of Medicine, University of Oslo, Oslo, Norway; 3 Norwegian Institute of Public Health, Oslo, Norway; 4 Norwegian National Advisory Unit on Women’s Health, Oslo University Hospital, Oslo, Norway; Indiana University School of Medicine, UNITED STATES

## Abstract

**Objective:**

Our main objective was to investigate the association between duration of active labor and severe postpartum hemorrhage. We examined the effect of the total duration of active labor, the effect of each stage of active labor, and the gradient effect of duration of labor on severe postpartum hemorrhage.

**Methods:**

A case-control study was generated from a source population of all women admitted for delivery at Oslo University Hospital and Drammen Hospital in Buskerud municipality during the time period January 1, 2008 to December 31, 2011. The study population included all cases of severe postpartum hemorrhage (n = 859) and a random sample of controls (n = 1755). Severe postpartum hemorrhage was defined as postpartum blood loss ≥1500 mL or need for blood transfusion. Prolonged labor was defined as duration of active labor >12 hours according to the definition of the World Health Organization. We used logistic multivariable regression in the analysis.

**Results:**

We observed a significantly longer mean duration of labor in women who experienced severe postpartum hemorrhage compared to controls (5.4 versus 3.8 hours, p<0.001). Women with severe postpartum hemorrhage also had a longer duration of all stages of active labor compared to controls. The association between the duration of active labor and severe postpartum changed from a linear dose-response association to a threshold association after adjusting for augmentation with oxytocin, induction of labor, primiparity, and fever during labor. Compared to controls, women with severe postpartum hemorrhage were more likely to have a prolonged labor >12 hours (adjusted odds ratio = 2.44, 95% confidence interval: 1.69–3.53, p< 0.001).

**Conclusion:**

Prolonged active labor (duration >12 hours) was associated with severe postpartum hemorrhage. Increased vigilance seems required when the labor is prolonged to reduce the risk of severe postpartum hemorrhage.

## Introduction

Severe postpartum hemorrhage (PPH) contributes substantially to maternal morbidity in high-income countries, causing >50% of all severe maternal morbidity [1]. Recent studies have shown an increasing trend in PPH, but the causes for this increase are still uncertain [2–4]. Obstetric interventions such as inductions of labor and oxytocin during labor, are increasingly common and believed to influence both the duration of labor and the risk for severe PPH [5–10]. By comparing labor patterns in the 1960s with a modern cohort, a study by Laughon et al [11] found an increased duration of the first stage of labor. They observed an increased use of obstetric interventions such as oxytocin, epidurals, and induction of labor in addition to the mothers being both older and of greater body mass index (BMI). The increase in labor duration persisted after adjusting for maternal and pregnancy characteristics, suggesting that changes in obstetric practices may be the primary reason for the increase. Normal labor has been defined as when an infant is born within 12 hours of active labor [12]. The World Health Organization (WHO) defines a prolonged active phase as regular painful contractions for more than 12 hours after cervical dilation of ≥4 cm [13].

Few studies have explored the association between the total duration of active labor and PPH. Several authors have reported that a prolonged second stage of labor is associated with PPH [14–16], but previous studies report conflicting results on whether the total duration of active labor increases the risk of PPH. One study looking at the first stage in women with induced labors reported an increased risk of PPH [17], while another study looking at both first and second stage in low-risk nulliparous women reported an association only with the second stage of labor [18]. The International PPH Collaborative Group [19] has recommended that prolonged labor should be among the potential risk factor for PPH investigated in future research.

In this study, our aim was to assess the association between the duration of active labor and severe PPH in women with intended vaginal deliveries.

## Materials and methods

**Fig 1:**
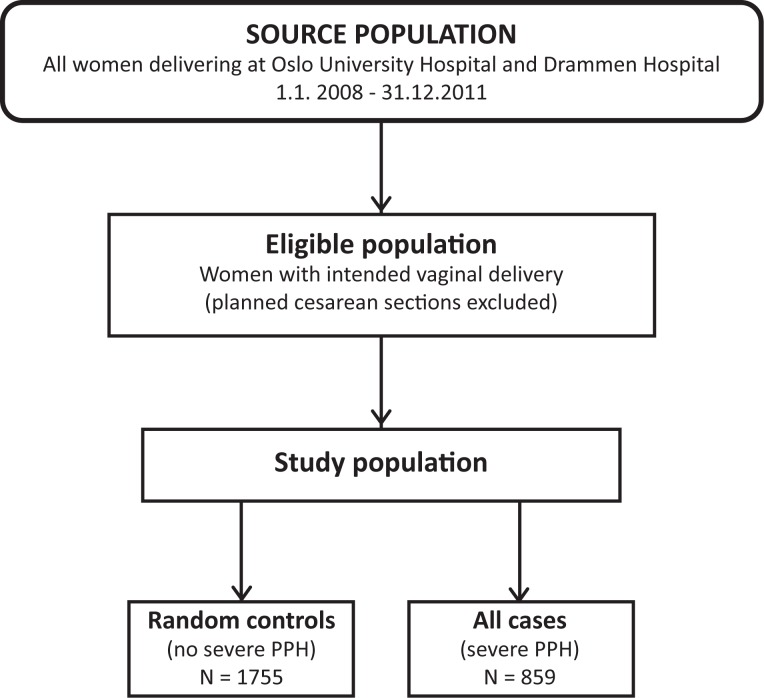
Selection of study subjects. PPH = postpartum hemorrhage.

The source population was all women admitted for labor at gestational week ≥23 at two University Hospitals in Oslo (Ullevaal and Rikshospitalet) and Drammen Hospital in Buskerud municipality during the period January 1, 2008 to December 31, 2011. After exclusion of women with planned caesarean deliveries, we extracted the study population, which included all cases of severe PPH (n = 859) and a random sample of controls without severe PPH (n = 1755) (Fig 1).

A de facto power analysis indicated that we needed at least 646 cases and 1292 controls considering two controls per case, type I error of 5%, power of 80%, frequency of prolonged labor in control subjects of 3%, and cases being twice as likely to be exposed to prolonged labor compared to controls (odds ratio (OR) = 2.00).

We defined severe PPH as postpartum blood loss ≥1500 mL within 24 hours of delivery of the infant or the need for blood transfusion for excessive bleeding at the time of delivery. The attending physician or midwife estimated the blood loss visually in all three hospitals. Blood transfusion for excessive bleeding was defined as a blood transfusion given for a likely PPH ≥1500 mL due to clinical symptoms and signs of anemia or hemodynamic decompensation after delivery. In Norway, National Guidelines in Obstetrics [20] recommends blood transfusion when the blood loss exceeds 1500 mL in an emergency situation with ongoing postpartum bleeding or if the measured hemoglobin is <7 g/dl after delivery. We excluded women who received a blood transfusion because of postpartum anemia, without evidence of excessive hemorrhage.

Further, we defined the total duration of active labor as the time from the cervix dilation of 3–4 cm and regular contractions until the infant was delivered. The active first stage was defined from the start of active labor (3–4 cm cervix dilation and regular contractions) until the cervix was fully dilated to 10 cm. Passive second stage was defined as the time from the cervix was dilated 10 cm until start of pushing; and the active second stage from the start of pushing until the infant was delivered. Prolonged labor was defined as the duration of active labor >12 hours, according to WHO`s definition [13].

We considered information on major risk factors for severe PPH and selected potential confounders on the association between prolonged labor and severe PPH. Selected variables were; maternal age, body mass index (BMI), birthweight of the infant, parity, previous cesarean, previous severe PPH, uterine fibromas, assisted reproductive technology (in vitro fertilization [IVF]/intracytoplasmic sperm injection [ICSI]), multiple pregnancy, anemia (Hb <9.0 g/dl) at the start of pregnancy, induction of labor, oxytocin augmentation during labor, and fever (>38°C) during labor. The continuous variables of maternal age, BMI, and birthweight were also dichotomized; BMI >30 kg/m2 according to the definition of obesity, birthweight ≥4500 g according to the definition of fetal macrosomia, and a distribution-related cutoff for maternal age >35 years according to the upper quartile.

Registration of patient data was based on information from the hospitals’ 1) medical records, 2) maternity databases (Obstetrix® and Partus®), and 3) birth suite protocols. Two experienced gynecologists reviewed all the medical records and all data were entered into a database built in EpiData Version 3.1 (EpiData Association, Odense, Denmark) by the first author. After registration was completed, we cleaned the database and outliers and categorization errors were checked against the medical records.

We investigated the association between duration of active labor and the occurrence of severe PPH. The other variables were of interest as potential confounders of the association between duration of labor and severe PPH. We used the OR and its 95% confidence intervals (CIs) to quantify this association. We performed univariate analyses to identify any association between the potential risk factors and severe PPH, followed by bivariate analyses to examine the effect of each variable on the association between prolonged labor and severe PPH. To control for multiple confounders, we used a multivariable logistic regression model [21]. In the multivariable analysis, we controlled for all variables causing a >10% change in the crude OR of the association between prolonged labor and severe PPH in the bivariate analyses. By splitting duration of labor into 4 categories of duration; < 4 hours, 4–7 hours, 7–12 hours, and >12 hours, we used a Mantel-Haenszel test of linear trend to investigate the dose-response effect for different exposure levels of duration of labor [22]. We then performed another multivariable logistic regression where we replaced the variable prolonged labor with the categorized variable and controlled for the same potential confounders as in the first regression model.

Finally, to compare the median duration at each stage of the active labor between cases and controls, we used a Mann-Whitney U-test.

We performed the statistical analyses with STATA 13.0 (StataCorp LP, College Station, TX, USA) and R version 3.2.2 (www.r-statistics.com). In reporting our case-control study, we used the Strengthening the Reporting of Observational Studies in Epidemiology (STROBE) statement guidelines [23]. The Regional Ethics Committee in the South-East Health region in Norway approved the study (reference number 2010/109a).

## Results

In this study, we found that a duration of active labor >12 hours was associated with severe PPH. The duration of labor was significantly longer in all stages of active labor in women suffering from severe PPH compared to controls (Table 1).

**Table 1:** Comparison of the median duration of labor in cases of severe postpartum hemorrhage versus controls, n = 2614.

	Cases (n = 859)	Controls (n = 1755)	
	**Hours (IQR)**	**Hours (IQR)**	**P-value**^**a**^
Active labor total	5.4 (2.9–9.0)	3.8 (2.0–6.7)	<0.001
Active 1^st^ stage	3.9 (1.9–6.7)	2.8 (1.3–5.0)	<0.001
	**Minutes (IQR)**	**Minutes (IQR)**	
Passive 2^nd^ stage	15 (0–60) min	10 (0–35) min	<0.001
Active 2^nd^ stage	32 (15–58) min	22 (10–45) min	<0.001

^a^Mann-Whitney U-test

IQR = interquartile range (25th - 75th percentiles); active labor total = from dilated cervix of 3–4 cm and regular contractions until infant is delivered; active 1^st^ stage = from 3–4 cm until 10 cm; passive 2^nd^ stage = from 10 cm until start of pushing; active 2^nd^ stage = from start of pushing until infant is delivered.

Among the cases of severe PPH, 507 women (59.0%) had a spontaneous vaginal delivery vs. 1358 women (77.3%) in the control group, 214 women (24.9%) vs. 252 (14.4%) had an instrumental vaginal delivery (forceps/ventouse), and 138 women (16.1%) vs. 145 (8.3%) had an in-labor cesarean delivery. Atony was the most common cause of severe PPH (62.3%), followed by retained placenta (24.6%). Moreover, surgical trauma during cesarean section was reported in 6.5% of the cases of severe PPH.

The characteristics of the study population are presented in Table 2. There were no clinically significant differences in median age, BMI, or infant birthweight between cases and controls. However, cases were more frequently nulliparous or anemic at the start of pregnancy, and more often achieved pregnancy through assisted reproductive technologies (IVF/ICSI) compared to controls. Moreover, they more frequently had previous severe PPH, multiple pregnancies, induction of labor, augmentation with oxytocin, and fever during labor.

**Table 2:** Clinical profile of the study population, n = 2614.

Risk factor	Cases (n = 859) n (%)	Controls (n = 1755) n (%)	OR (95% CI)	p-value
Nulliparous	519 (60.4)	876 (49.9)	1.53 (1.30–1.81)	<0.001
Previous cesarean	69 (8.0)	114 (6.5)	1.26 (0.92–1.72)	0.149
Previous severe PPH	48 (5.6)	13 (0.7)	7.93 (4.27-14-72)	<0.001
Uterine fibroma	23 (2.7)	19 (1.1)	2.51 (1.36–4.64)	0.003
IVF/ICSI-pregnancy	78 (9.1)	62 (3.5)	2.72 (1.93–3.85)	<0.001
Multiple pregnancy	57 (6.6)	27 (1.5)	4.55 (2.86–7.25)	<0.001
Anemia in start of pregnancy	56 (7.1)	35 (2.1)	3.56 (2.32–5.49)	<0.001
Induction of labor	336 (39.1)	381 (21.7)	2.32 (1.94–2.77)	<0.001
Oxytocin augmentation	583 (67.9)	796 (45.3)	2.55 (2.14–3.02)	<0.001
Fever (>38°C in labor)	72 (8.4)	56 (3.2)	2.76 (1.94–3.98)	<0.001
Duration of labor >12hours	89 (10.4)	53 (3.0)	3.71 (2.61–5.27)	<0.001
Age >35 years	208	375	1.76 (0.96–1.43)	0.101
BMI >30 kg/m^2^	132	216	1.29 (1.02–1.63)	0.031
Birthweight ≥4500g	46	53	1.82 (1.21–2.71)	0.004
	**Median (IQR)**	**Median (IQR)**		
Age (years)	32 (29–35)	32 (28–35)	1.52 (0.93–2.51)	0.098
BMI (kg/m^2^)	23 (21–26)	23 (21–26)	1.46 (0.88–2.41)	0.140
Birthweight (grams)	3626 (3246–3980)	3498 (3170–3850)	1.86 (1.21–2.86)	0.005

BMI = body mass index; IQR = interquartile range (25th - 75th percentiles); IVF/ICSI = in vitro fertilization/intra-cytoplasmic sperm injection; PPH = postpartum hemorrhage; PROM = premature rupture of membranes.

Women with severe PPH were nearly 4 times more likely to have a prolonged labor compared to controls without severe PPH (crude OR = 3.71; 95% CI 2.61–5.27). After adjusting for induction of labor, augmentation with oxytocin, primiparity, and fever during labor in the multivariable regression analysis, women with severe PPH had a 2.4-fold increased risk of prolonged labor compared to controls (adjusted OR = 2.44; 95% CI 1.69–3.53) (Table 3).

**Table 3:** Multivariable analysis on the association between duration labor >12 hours and severe postpartum hemorrhage, n = 2614.

Risk factor*	Unadjusted OR (95% CI)	Adjusted OR** (95% CI)	p-value
Duration of labor >12 hours	3.71 (2.61–5.27)	2.44 (1.69–3.53)	<0.001
Oxytocin augmentation	2.55 (2.14–3.02)	1.89 (1.56–2.29)	<0.001
Induction	2.32 (1.94–2.77)	1.95 (1.62–2.36)	<0.001
Primiparity	1.53 (1.30–1.81)	1.14 (0.92–1.34)	0.248
Fever during labor	2.76 (1.94–3.98)	1.88 (1.29–2.74)	<0.001

*All risk factors are dichotomized yes/no.

**Adjusted for all variables in the table.

**Fig 2:**
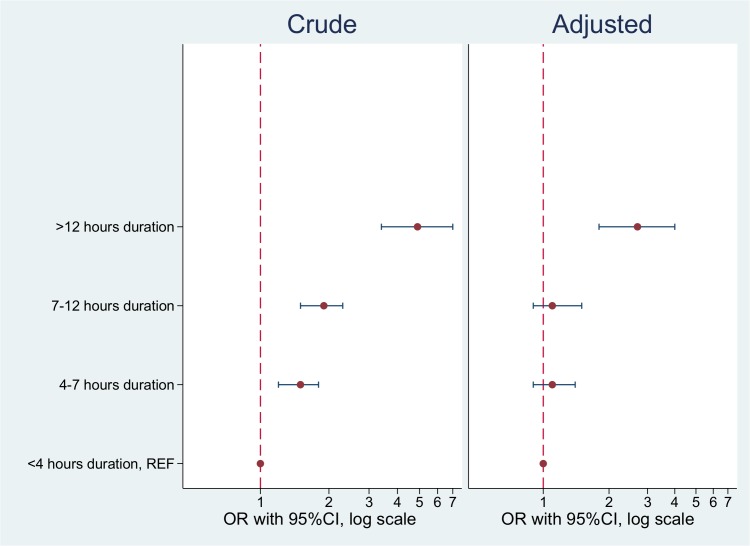
Gradient effect of the duration of labor on severe postpartum hemorrhage, n = 2614. Adjusted for augmentation with oxytocin, induction of labor, primiparity, and fever during labor. OR = odds ratio; CI = confidence interval.

Furthermore, a significant Mantel-Haenszel chi-square test for linear trend (χ^2^ = 84.235, p<0.001) indicated a linear dose-response association between the duration of labor and severe PPH; duration of labor < 4 hours (reference), 4–7 hours: crude OR = 1.5 (95% CI 1.22–1.85), 7–12 hours: crude OR = 1.9 (95% CI 1.50–2.28), and >12 hours: crude OR = 4.9 (95% CI 3.4–7.0). However, after adjusting for induction of labor, augmentation with oxytocin, primiparity, and fever during labor, the association changed from a linear dose-response association to a threshold association; duration of labor < 4 hours (reference), 4–7 hours: aOR = 1.11 (95% CI 0.88–1.40), 7–12 hours: aOR = 1.11 (95% CI 0.85–1.45), and >12 hours: aOR = 2.66 (95% CI 1.76–4.02) (Fig 2).

## Discussion

In this study, we found that a duration of active labor of >12 hours was associated with an increased risk of severe PPH. Compared to controls, women with severe PPH more often had a prolonged labor >12 hours (aOR = 2.44 (95% CI 1.69–3.53). Both the first and the second stage of labor were longer in cases with severe PPH compared to controls, with 3.9 versus 2.8 hours and 47 versus 32 minutes, respectively. Furthermore, the effect of the duration of labor on severe PPH changed from a linear dose-response association to a threshold association after adjusting for potential confounding factors.

The strengths of the study included sufficient statistical power to test our main hypothesis. Meticulously reading the patients’ medical records enabled us to provide more complete data as compared to registry studies. The potential for selection bias is minimal because cases and random controls were derived from the same source population during the same time period, and the eligibility criteria applied equally to both groups. There is a probability that some cases were misclassified. Visual estimation was the usual practice to estimate blood loss in our study and might have been underestimated; however, we had further data in the medical records to assess severe PPH by need for a blood transfusion. We believe the possibility of information bias was minimal because exposure information was obtained from patients’ medical records and the resident obstetrician/midwife was unaware of our research questions. In this situation, misclassification of exposure or disease status was more likely non-differential, which could have created bias toward the null effect [21]. As for confounding, we aimed to include major risk factors for severe PPH in the protocol, but there is always a possibility of unmeasured confounding in observational studies. Our results cannot necessarily be generalized to a wider population because our source population was restricted to an urban setting; three hospitals in or close to Oslo.

Our study shows an association between labor duration in all stages of active labor and severe PPH. In a registry based study of risk factors for PPH >1000 mL, Stones et al [24] reported an increased risk of severe PPH if the labor lasted more than 12 hours (aOR = 2.0; 95% CI 1.4–2.9). Another larger registry based study of risk factor for PPH [25] only found a non-significant association with labor lasting >12 hours and severe PPH ≥1500 mL (aOR = 1.9; 95% CI 0.7–5.6). Le Ray et al [18] reported a significantly longer active second stage in women who experienced severe PPH, while no increased risk of PPH was found with a prolonged active first stage. However, their study included only 69 low-risk nulliparous women, representing a subgroup of delivering women. Our study included 859 high- and low-risk women of all parities who experienced severe PPH. In a study examining the first stage of labor, Cheng et al [17] found an increased risk of PPH with a prolonged first stage of labor, but this study included only women with induced labors.

While previous study results are conflicting regarding the association between prolonged first stage of labor and PPH, several studies have reported an increased risk of PPH with a prolonged second stage of labor [14–16]. However, it is reasonable to assume that the total duration of active labor, and not only the second stage, impacts on the risk of severe PPH. A long-lasting labor, including a prolonged first stage, may increase the risk of PPH by causing uterine atony in the third stage (after the infant is delivered). Uterine atony occurs when the relaxed myometrium fails to constrict the uterine blood vessels. Regular contractions over several hours of labor will exhaust the uterine muscles and thereby reduce their contractility over time, causing uterine dysfunction.

Causes of uterine dysfunction before the onset of active labor, such as uterine fibromas, overdistended uterus, scarred uterus, and infection, may result in delay of all stages of labor and thereby cause PPH. However, the increased risk by having a prolonged active labor was not explained by these risk factors in our study. Focusing on the second stage of labor to reduce the risk of PPH is important, but an increased vigilance when it comes to the total duration of active labor seems warranted as well.

Oxytocin is widely used for augmentation of labor and has been associated with PPH in previous studies [10, 26]. Oxytocin can be protective by shortening the labor time and consequently the risk of severe PPH. However, the drug can increase the risk of PPH after prolonged exposure by desensitizing the uterine oxytocin receptors [27–29], resulting in loss of contractile function and uterine atony. Induction of labor has also been shown to be associated with PPH in previous studies [5, 25]. An increasing rate of labor inductions have been reported [30] and inductions are often done on request and hence without any strict medical indication [31, 32]. A reduction in the risk of PPH may be achieved by changing to evidence-based treatment protocols involving both induction and augmentation of labor. Infection during labor has been reported to increase the risk of PPH [24, 33, 34] and may predispose for uterine atony and PPH by contributing to uterine dysfunction or inertia [35].

As studies have reported increased labor duration among pregnant women over the past decades [11], prolonged duration of labor may be part of the explanation for the observed increased trend in PPH in high-income countries.

In conclusion, the results of this study underline the importance of being aware that prolonged active labor >12 hours is associated with an increased risk of severe PPH. All interventions that prolong labor will add to the risk of severe PPH and should be used with caution.
